# Trench FinFET Nanostructure with Advanced Ferroelectric Nanomaterial HfZrO_2_ for Sub-60-mV/Decade Subthreshold Slope for Low Power Application

**DOI:** 10.3390/nano12132165

**Published:** 2022-06-23

**Authors:** Siao-Cheng Yan, Chen-Han Wu, Chong-Jhe Sun, Yi-Wen Lin, Yi-Ju Yao, Yung-Chun Wu

**Affiliations:** Department of Engineering and System Science, National Tsing Hua University, Hsinchu 30013, Taiwan; jeff910029@gmail.com (S.-C.Y.); jackywu0512@gmail.com (C.-H.W.); jj199658@gmail.com (C.-J.S.); da373816w@gmail.com (Y.-W.L.); yiju0410@gmail.com (Y.-J.Y.)

**Keywords:** FinFET, FeFET, hafnium zirconium oxide, steep slope, trench

## Abstract

Ferroelectric fin field-effect transistors with a trench structure (trench Fe-FinFETs) were fabricated and characterized. The inclusion of the trench structures improved the electrical characteristics of the Fe-FinFETs. Moreover, short channel effects were suppressed by completely surrounding the trench channel with the gate electrodes. Compared with a conventional Fe-FinFET, the fabricated trench Fe-FinFET had a higher on–off current ratio of 4.1 × 10^7^ and a steep minimum subthreshold swing of 35.4 mV/dec in the forward sweep. In addition, the fabricated trench Fe-FinFET had a very low drain-induced barrier lowering value of 4.47 mV/V and immunity to gate-induced drain leakage. Finally, a technology computer-aided design simulation was conducted to verify the experimental results.

## 1. Introduction

Since no additional power supply is available, power consumption is the major concern for conventional complementary metal–oxide–semiconductor (CMOS) based integrated circuits for wearable applications. The wearable devices are supposed to operate in an intermittent mode; thus, the power consumption of the wearable devices will be dominated by standby leakage power [1,2]. To suppress current leakage and maintain the drive current and dynamic performance, the subthreshold swing (SS) must be reduced to maintain the high on–off current ratio. However, the Boltzmann limitation of SS [3,4] is inevitable for traditional metal–oxide–semiconductor field-effect transistors (MOSFETs) and strongly limits the reduction of power consumption. For Internet of Things (IoTs), wearable electronics, and next-generation technology node applications, electronic components with ultralow power consumption and high performance must be developed. Therefore, ferroelectric field-effect transistors (FeFETs) with doped HfO_2_ thin films [5,6,7] as the gate insulator have become a promising solution for overcoming the fundamental thermionic limit of SS (≈60 mV/dec) of MOSFETs. The HfO_2_ thin films represent the state-of-the-art gate insulator for essentially all high-k metal-gate process nodes. By modifying the gate insulator to ferroelectric doped HfO_2_, numerous experimental FeFETs have achieved an SS of <60 mV/dec due to the negative capacitance (NC) effect [8,9,10,11,12,13,14]. Accordingly, nanomaterials doped with HfO_2_ are suitable for devices for low power applications such as wearable electronics and IoTs.

The ferroelectric fin field-effect transistor (Fe-FinFET) is the most promising candidate for near-future applications, which combines the state-of-the-art technology node and the ferroelectric gate insulator. To further improve its performance, an Fe-FinFET with a trench structure (trench Fe-FinFET) is proposed in this work. Most FETs with a trench structure produced to date [15,16,17,18] have been designed as junctionless, ultra-thin channel transistors to ensure that the channel is fully depleted and to suppress short channel effects (SCEs). However, this design may result in degraded carrier mobility [19] and lower on current (*I*_ON_). In this work, we integrated a trench structure into a Fe-FinFET to suppress SCEs and gain the advantages of the trench structure. Furthermore, a trench channel with higher thickness and an inversion-mode-type FET were designed to prevent the aforementioned drawbacks. Technology computer-aided design (TCAD) simulations were also conducted to verify the experimental results and understand the device physics.

## 2. Device Fabrication

**Figure 1:**
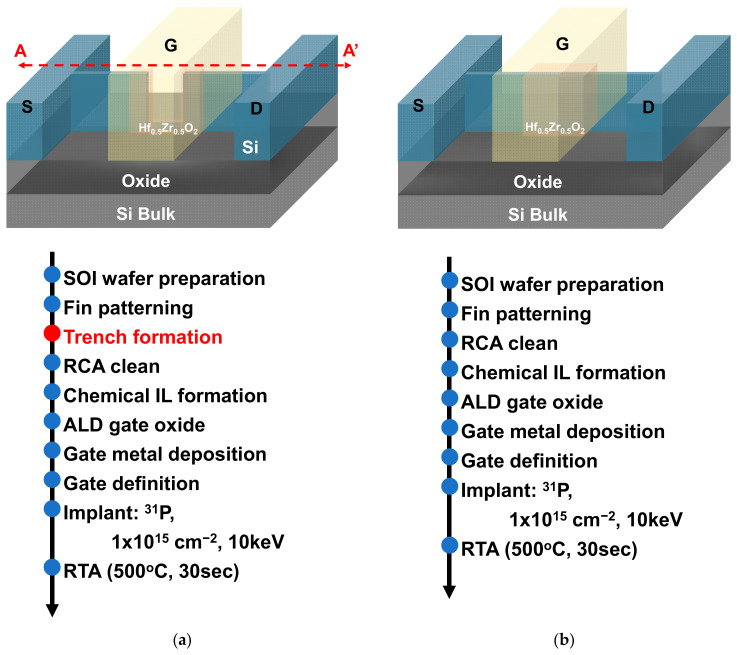
Schematics and process flows of (**a**) the trench Fe-FinFET and (**b**) conventional Fe-FinFET.

Figure 1a,b present the schematics and the process flows of the trench Fe-FinFET and conventional Fe-FinFET, respectively. The trench Fe-FinFET devices have a trench structure channel, which was realized with an additional etching after fin shape definition. Both the trench Fe-FinFET and Fe-FinFET devices were fabricated on p-type 65-nm-thick monocrystalline silicon/145-nm-thick buried SiO_2_/Si substrate on 8-inch silicon-on-insulator wafers. First, the active region with a fin width (*F*_W_) of 40 nm was defined by electron-beam lithography (EBL), and the fin shape was transferred by transformer-coupled plasma (TCP) etching. The etch process condition for Si was optimized with HBr flow of 450 sccm, O_2_ flow of 2 sccm, chamber pressure of 10 mTorr, TCP RF power of 700 W, and bias RF power of 80 W.

For the trench Fe-FinFET devices, the trench structure with a length of 100 nm was patterned with an additional mask, and the Si was subsequently thinned to 14 nm by anisotropic etching (Figure 3). After RCA cleaning, a chemical oxide interfacial layer (IL) was formed with H_2_O_2_, and a 5-nm-thick HfZrO_2_ (HZO) ferroelectric layer was deposited by atomic layer deposition at 250 °C with supercycles of layer-by-layer HfO_2_ and ZrO_2_. Hf(N(CH_3_)_2_)_4_ (TDMAH), Zr(N(CH_3_)_2_)_4_ (TDMAZ), and O_2_ plasma were used as the Hf precursor, Zr precursor, and oxygen source, respectively. Subsequently, an 80-nm-thick TaN layer was deposited as the gate electrode using ULVAC ENTRON W200 sputter (ULVAC, Chigasaki, Japan) with a DC power of 300 W, Ar flow of 20 sccm, and N_2_ flow of 2 sccm. The gate region with a series of gate lengths (*L*_G_) from 250 to 400 nm was patterned with EBL and TCP etching. The etch process condition for TaN was with Cl_2_ flow of 70 sccm, SF_6_ flow of 30 sccm, chamber pressure of 10 mTorr, TCP RF power of 600 W, and bias RF power of 100 W. After gate patterning, self-aligned implantation with P at a dosage of 1 × 10^15^ cm^−2^ and 10 keV was performed to form the N+ source (S) and drain (D). Finally, the ferroelectric layer crystallization and dopant activation were simultaneously performed by rapid thermal annealing (RTA) at 500 °C for 30 s.

## 3. Results and Discussion

**Figure 2:**
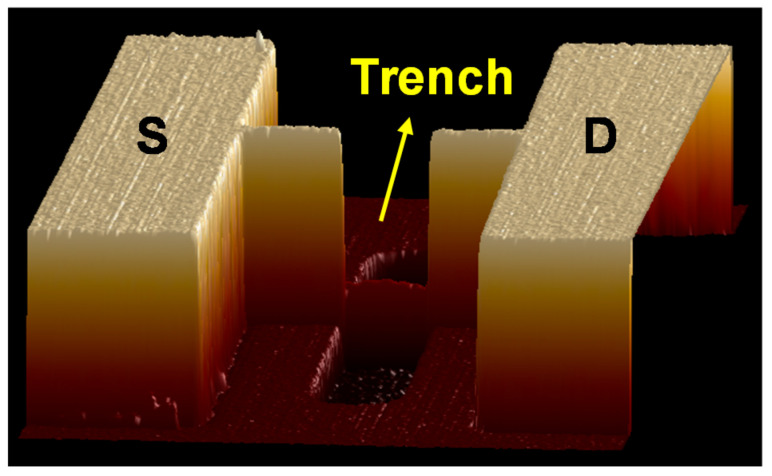
Three-dimensional AFM image of the active region and trench of a fabricated trench Fe-FinFET.

**Figure 3:**
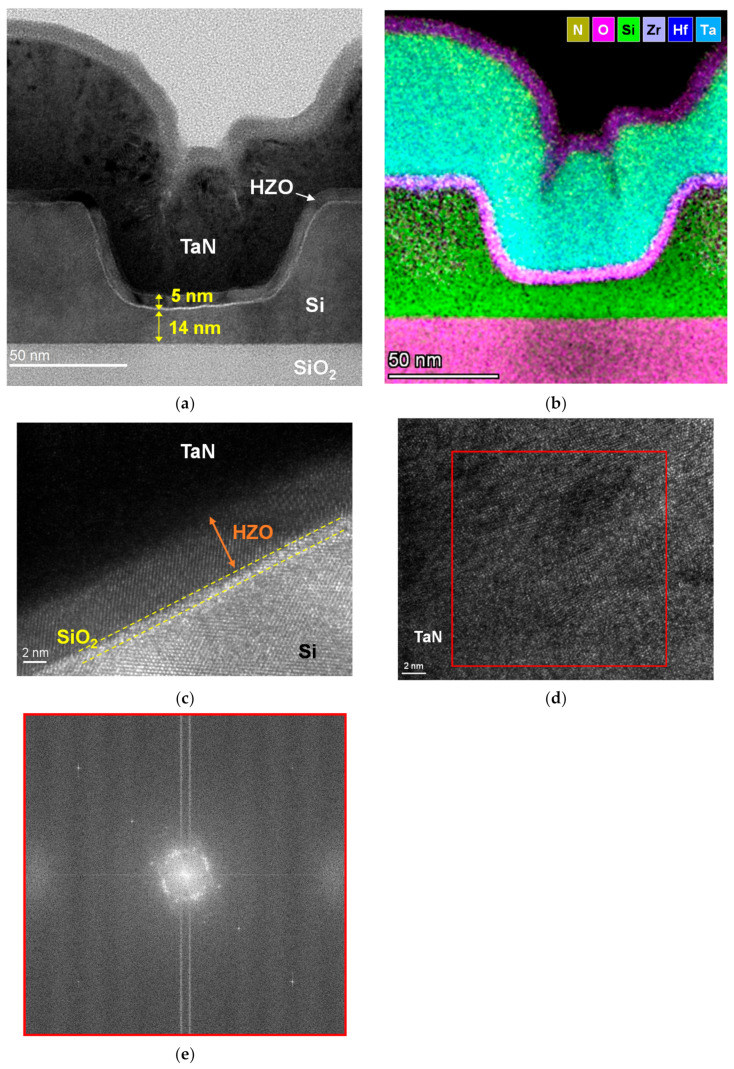
(**a**) TEM image of a fabricated Fe-FinFET device with 14-nm-thick trench, *L*_T_ of 100 nm, *L*_G_ of 250 nm, and HZO thickness of 5 nm. (**b**) Two-dimensional energy-dispersive X-ray spectroscopy mapping of the element distribution. (**c**) Enlarged view of the HZO/SiO2/Si interface. (**d**) Enlarged view of the TaN gate. (**e**) Diffraction pattern of the TaN layer.

Figure 2 presents a three-dimensional atomic force microscopy (AFM) (Bruker, Billerica, MA, USA) image of the active region with the trench structure of a fabricated device. The AFM image reveals that the Si surface maintained its smoothness after trench etching. Figure 3a presents an A–A’ cross-sectional transmission electron microscopy (TEM) (Thermo Scientific, Waltham, MA, USA) image of the fabricated trench Fe-FinFET device. Figure 3b presents the two-dimensional energy-dispersive X-ray spectroscopy mapping of the element distribution for the fabricated device. Figure 3c presents the zoomed in TEM image of the HZO/SiO_2_/Si interface. As shown in the TEM image, the ferroelectric HZO layer was well crystallized after RTA. The minimum thickness of Si in the trench was 14 nm, and the thickness of the HZO layer was 5 nm; these layers were separated by a chemical oxide IL with thickness approximately 1 nm. To ensure that the trench channel could be completely wrapped by the gate electrode to achieve better gate control ability [17], the trench length (*L*_T_) was set to 100 nm, and *L*_G_ was varied to be between 250 and 400 nm. Figure 3d,e present the enlarged view of the TaN gate and the diffraction pattern of TaN, respectively. The TEM image and diffraction pattern indicate that the TaN gate was polycrystalline, and the resistivity of TaN was approximately 300 µΩ∙cm, characterized by the four-probe method.

**Figure 4:**
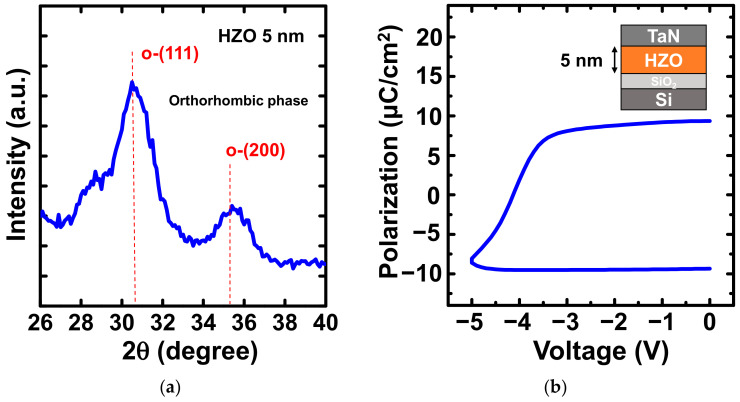
(**a**) The GIXRD pattern of 5-nm-thick HZO shows the orthorhombic phase in HZO film. (**b**) *P*–*V* characteristic for the MFIS capacitor with 5 nm HZO.

To prove the ferroelectric behavior of the 5-nm-thick ferroelectric layer, grazing incident X-ray diffraction (GIXRD) (PANalytical, Malvern, United Kingdom) and positive-up-negative-down (PUND) measurement were performed. Figure 4a presents the GIXRD analysis of the 5-nm-thick HZO film annealed at 500 °C for 30 s. The GIXRD pattern of ALD HZO showed strong peaks at approximately 30° and 35° that corresponded to the ferroelectric orthorhombic phase with space group Pbc2_1_ [20]. Figure 4b presents the polarization–voltage (*P*–*V*) curve measured by the PUND method for the metal/ferroelectric/insulator/semiconductor (MFIS) capacitor with 5 nm HZO after RTA at 500 °C for 30 s, which was the same condition as used for the fabricated devices. It is worth mentioning that under PUND measurement, applying positive pulses to the TaN top electrode for positive polarization measurement would create a depletion region generated from the p-type semiconductor and cause a voltage drop in the Si substrate [21]. Therefore, the actual voltage and polarization response for the positive polarization cannot be truly reflected. To prevent the depletion effect, only the negative polarization measurement was considered, as shown in Figure 4b. The negative remanent polarization (*P*_r_) for the MFIS capacitor was 9.34 µC/cm^2^, and the detected *P*_r_ in the MFIS capacitor was attributed to the non-centrosymmetric o-phase.

**Figure 5:**
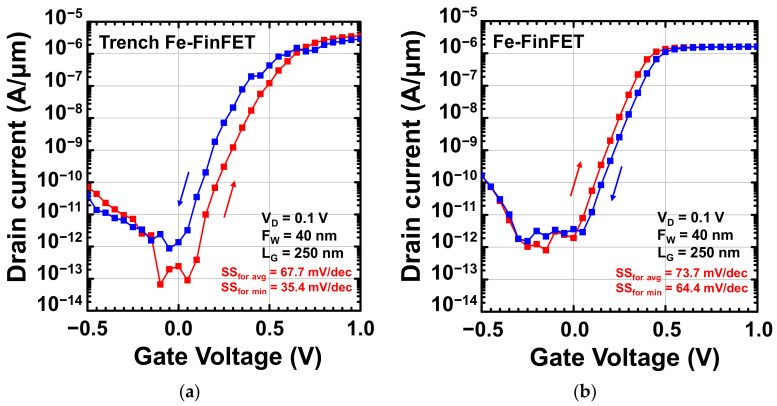
Transfer *I*_D_–*V*_G_ curves (*L*_G_ = 250 nm, *F*_W_ = 40 nm, and *V*_D_ = 0.1 V) of fabricated (**a**) trench and (**b**) conventional Fe-FinFETs, including forward and reverse sweeps. The sweep directions are indicated by the arrows on the plot.

Figure 5a,b present the transfer *I*_D_–*V*_G_ curves of the fabricated trench Fe-FinFET and Fe-FinFET, respectively. The *I*_D_–*V*_G_ curves, including the forward and reverse sweeps, were determined for the Fe-FinFETs with *F*_W_ = 40 nm and *L*_G_ = 250 nm at *V*_D_ = 0.1V, and *I*_D_ was normalized by the fin width (*F*_W_). The average SS (SS_avg_) was 67.7 mV/dec over the whole subthreshold region, and a minimum SS in the forward sweep (SS_for min_) of 35.4 mV/dec was achieved with the trench Fe-FinFET. Because the trench structure produces a strong NC effect, the trench Fe-FinFET has a much smaller minimum SS value than the conventional Fe-FinFET does. Furthermore, the experimental results indicated that the trench Fe-FinFET also has a higher *I*_ON_ and lower off current (*I*_OFF_) than the conventional Fe-FinFET. A high on–off current ratio of 4.1 × 10^7^ in the forward sweep was measured for the trench Fe-FinFET, and the *I*_OFF_ of the trench Fe-FinFET was almost one order of magnitude smaller than that of the conventional Fe-FinFET. Despite the SS_avg_ extracted from the whole range of subthreshold region being approximately 70 mV/dec for the trench Fe-FinFET, the steep slope region still results in a lower off current and a larger on–off current ratio. Notably, when applying the gate voltage from negative to positive on the gate of an n-type transistor with a ferroelectric layer, the dipoles in the ferroelectric layer would flip down and attract the electrons to the channel, therefore reducing the *V*_T_ value [22]. The reverse sweep might be the left shift. However, the charge trapping effect may dominate and compensate for the *V*_T_ shift of ferroelectric polarization switching and result in a clockwise hysteresis, as shown in the conventional Fe-FinFET in Figure 5b. The electric field enhancement of the trench Fe-FinFET attributed to the trench structure benefits dipole flipping and the ferroelectric behavior would dominate, hence causing a counterclockwise hysteresis, as shown in Figure 5a.

**Figure 6:**
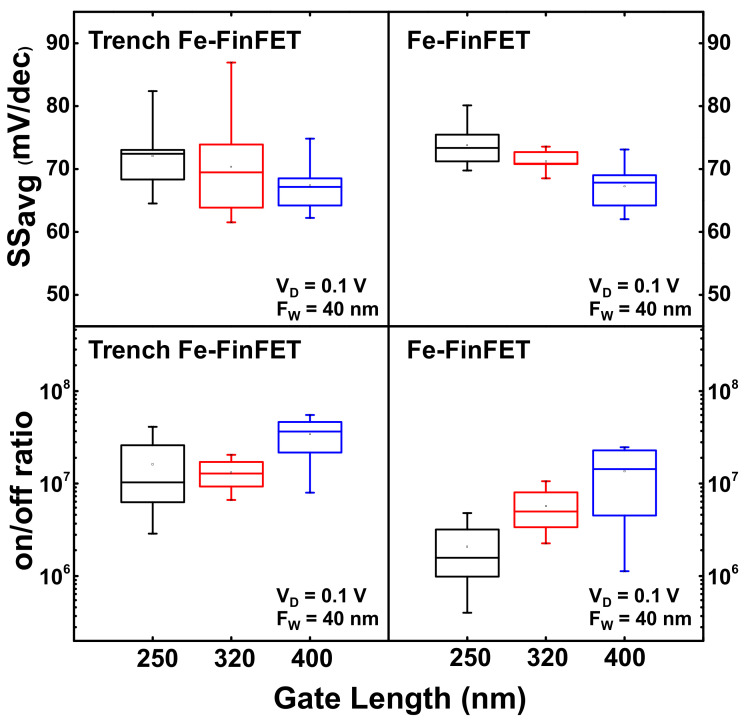
Statistical analysis of SS_avg_ and on–off current ratio with *L*_G_ = 250, 320, and 400 nm for the trench Fe-FinFET and the conventional Fe-FinFET.

For a more complete comparison of the electrical characteristics between the trench Fe-FinFET and conventional Fe-FinFET, the statistics of the SS_avg_ values and on–off current ratio of the fabricated devices with *L*_G_ = 250, 320, and 400 nm are shown in Figure 6. The SS_avg_ of the trench Fe-FinFET was slightly lower than that of the conventional Fe-FinFET because of the better gate control ability. Furthermore, the SS_avg_ decreased as the *L*_G_ varied from 250 to 400 nm for both the trench Fe-FinFET and conventional Fe-FinFET. This is because of the larger *L*_G_ dimensions, and the lower influence of the SCEs on the devices, therefore leading to a smaller SS_avg_ value. However, the trench Fe-FinFET devices had a steep slope region below 60 mV/dec because of the stronger NC effect. On the other hand, the conventional Fe-FinFET devices exhibited SS_min_ values of around 60 mV/dec. The steep slope region of the trench Fe-FinFET caused a lower off current and was reflected in the on–off current ratio. Most of the trench Fe-FinFET devices exhibited a larger on–off current ratio at *V*_D_ = 0.1 V than the conventional Fe-FinFET devices under the same *L*_G_ dimension. Furthermore, despite the large *L*_G_ dimension that would decrease the on current, the on–off current ratio still slightly increased with *L*_G_ varying from 250 to 400 nm, both in the trench Fe-FinFET and conventional Fe-FinFET. The larger on–off current ratio in large *L*_G_ devices can be attributed to the lower off current.

**Figure 7:**
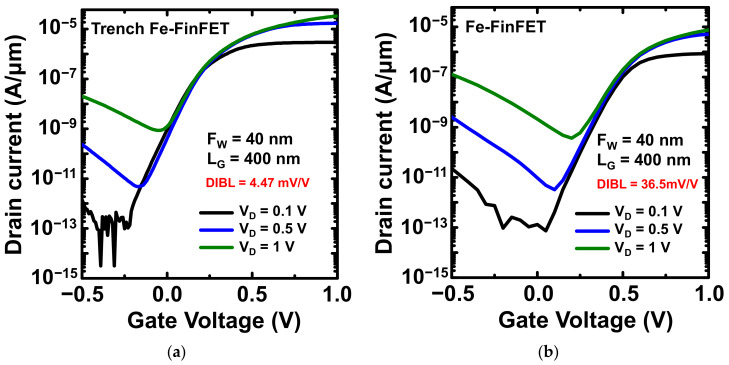
*I*_D_–*V*_G_ curves (*L*_G_ = 400 nm, *F*_W_ = 40 nm) for *V*_D_ = 0.1, 0.5, and 1 V for fabricated (**a**) trench and (**b**) conventional Fe-FinFETs. *V*_T_ was determined at a constant current of 10^−7^ A/µm.

**Figure 8:**
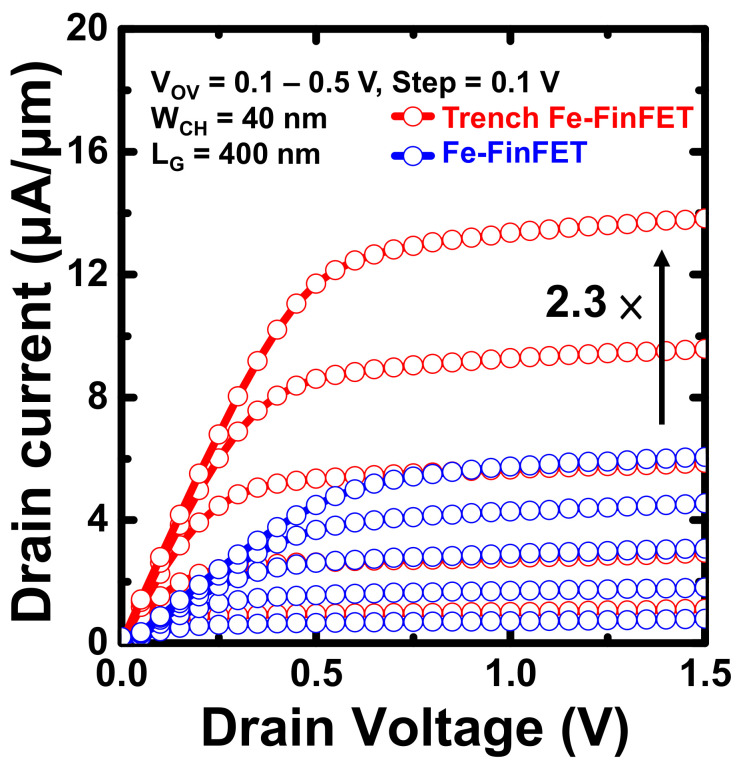
*I*_D_–*V*_D_ curves of a fabricated trench Fe-FinFET and a fabricated Fe-FinFET with *L*_G_ = 400 nm and *F*_W_ = 40 nm at *V*_OV_ = 0.1–0.5 V.

Figure 7a,b present the *I*_D_–*V*_G_ curves at *V*_D_ = 0.1, 0.5, and 1 V for the fabricated trench Fe-FinFET and Fe-FinFET, respectively; both Fe-FinFETs had *L*_G_ = 400 nm. *V*_T_ was determined at a constant current of 10^−7^ A/µm. Both devices had high immunity to drain-induced barrier lowing (DIBL) values of 4.47 and 36.5 mV/V for the trench and conventional Fe-FinFETs, respectively. The FinFET is a structure well known for suppressing SCEs [23]. In this work, a thin Si trench was wrapped around the gate electrode of the trench Fe-FinFET, further preventing the electric field from penetrating from the drain to the source and therefore reducing the DIBL. Moreover, the trench Fe-FinFET device has lower *I*_OFF_ at various *V*_D_ values and also has high immunity to gate-induced drain leakage. Figure 8 presents the output *I*_D_–*V*_D_ characteristics of the trench and conventional Fe-FinFET devices both with *L*_G_ = 400 nm at *V*_OV_ (*V*_GS_−*V*_T_) from 0.1 to 0.5 V with a step of 0.1 V for comparison. The trench Fe-FinFET had a saturation current approximately 2.3 greater than the conventional Fe-FinFET at *V*_OV_ = 0.5 V. This higher *I*_ON_ can be attributed to the higher electron velocity and electron density in the trench, especially around the valleys of the trench [16].

**Figure 9:**
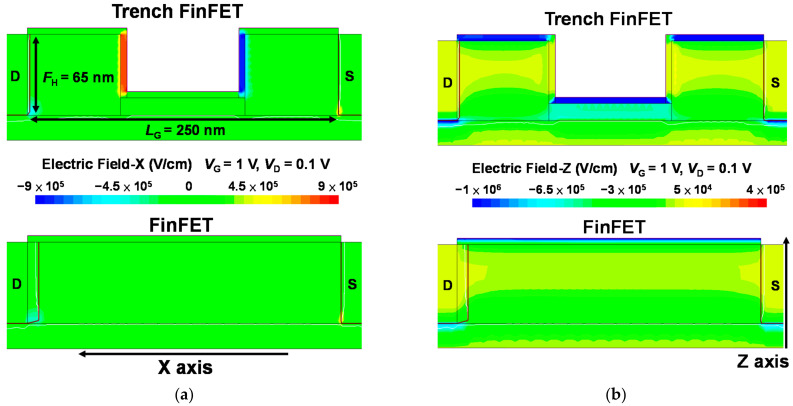
TCAD simulation results for the electric field in the (**a**) *x* and (**b**) *z* directions for the Si-trench FinFET and Si FinFET. The trench FinFET has a much stronger electric field in the gate insulator than the conventional FinFET does.

**Figure 10:**
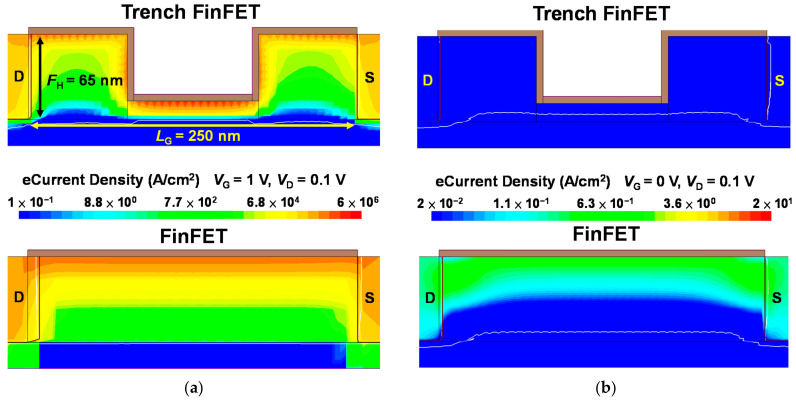
TCAD simulation results for the electron current density in (**a**) the strong inversion region and (**b**) surface depletion region of an Si trench FinFET and an Si FinFET. The trench FinFET has greater electric current density in the strong inversion region and lower electric current density in the surface depletion region.

TCAD simulations (Synopsys, Mountain View, CA, USA) were performed to further understand how the trench structure improves the electrical characteristics. The simulation was designed in accordance with the experimental data illustrated in Figure 3 and the corresponding FinFET structure (*L*_G_ = 250 nm and *F*_W_ = 40 nm). Figure 9a,b present the TCAD simulation results for the electric field of the Si trench FinFET and Si FinFET at *V*_G_ = 1 V and *V*_D_ = 0.1 V in the *x* and *z* directions, respectively. As depicted in the simulation results, the electric field in the gate insulator of the trench FinFET was much stronger than that in the conventional FinFET. This electric field enhancement occurs in the direction perpendicular to the gate electrode and gate insulator and induces fast dipole flipping [24]. This result is consistent with the low SS values of the trench Fe-FinFET due to the stronger NC effect. Figure 10 presents a comparison of the simulation results for the electron current density of the Si trench FinFET and Si FinFET in the strong inversion region (*V*_G_ = 1 V, *V*_D_ = 0.1 V) and surface depletion region (*V*_G_ = 0 V, *V*_D_ = 0.1 V).

The Si trench FinFET had an electron current density approximately 1.7 times greater than the conventional FinFET in the strong inversion region, and the much lower electron current density of the Si trench FinFET in the surface depletion region could be clearly observed. Because the current density includes the effects of carrier density, carrier velocity, and the electric field, simulations of current density are an intuitive method of understanding how a trench structure improves the electrical characteristics. In conclusion, the simulation results reveal the source of larger *I*_ON_ and lower *I*_OFF_ values, which correspond to the high on–off ratio achieved in the experimental results.

## 4. Conclusions

Fe-FinFETs with trench structures were fabricated and characterized. The fabricated trench Fe-FinFET had a steep minimum SS of 35.4 mV/dec due to a strong NC effect that was attributed to fast dipole flipping; this was in turn due to its high electric field strength across the ferroelectric layer of the trench structure. Moreover, compared with a conventional Fe-FinFET, the fabricated trench Fe-FinFET had better gate control ability, increased immunity to SCEs, and a higher on–off current ratio of 4.1 × 10^7^ due to its thin trench channel. Finally, these phenomena were verified by TCAD simulations, and the device physics was also discussed. The fabrication processes used to produce the trench Fe-FinFET are compatible with those used for Si-CMOS devices. With one simple process, the inclusion of the trench structures improved the electrical characteristics of the Fe-FinFETs. Thus, the as-fabricated trench Fe-FinFET is a promising candidate for ultralow-power applications and three-dimensional stacked integrated circuit applications.

## Data Availability

The data presented in this study are available on request from the corresponding author.
